# Editorial: Advancing digital mental health for youth

**DOI:** 10.3389/fdgth.2026.1828208

**Published:** 2026-03-30

**Authors:** Andreas Balaskas, Wanling Cai, Raymond R. Bond, Kevin Doherty

**Affiliations:** 1 School of Computer Science, University College Dublin, Dublin, Ireland; 2 School of Computing, Ulster University, Belfast, United Kingdom; 3 School of Information and Communication Studies, University College Dublin, Dublin, Ireland

**Keywords:** artificial intelligence, digital interventions, digital mental health, help-seeking, mental health literacy, mental health prevention, personalization, youth mental health

## 1 Introduction

Young people (aged 12–25) face a particularly high-risk developmental period. Not only do 75% of mental illnesses emerge before the age of 25 (1, 2), but the rising prevalence today increases the demand for support (3, 4). The rapid expansion of digital technologies is reshaping prevention, assessment, and intervention in youth mental health, offering new opportunities for accessible and cost-effective care (5–7). Digital tools increasingly deliver psychological support in everyday contexts, as recent advances in online platforms, app-based interventions, wearable devices, remote monitoring technologies, and AI applications present opportunities to provide personalized and accessible mental health resources (8–10) while at the same time raising new questions concerning the responsible adoption and adaptation of artificial intelligence (AI) technologies for mental health and wellbeing (11).

## 2 Research topic content

As global demand for services continues to exceed the available resources, digital approaches are increasingly viewed as key components of scalable systems of care. The accelerating integration of digital technologies into everyday life has fundamentally reshaped how young people experience, express, and manage their mental health. This Research Topic examines the opportunities and challenges of technological interventions for youth mental health, including emerging tools such as AI. The 13 contributions to *Advancing Digital Mental Health for Youth* highlight a diverse range of approaches to supporting youth mental health through digital technologies. As Topic Editors, we have sought to bring together studies surfacing and examining the central questions of prevention, assessment, intervention design and implementation in the context of this rapidly evolving technological field.

Recent advances highlight the growing role of adaptive and personalized approaches in digital mental health. Tong et al., present an AI-driven dynamic psychological measurement approach that recalibrates traditional anxiety and depression scales using daily behavioral and cognitive data from university students. The dynamic model outperformed static scoring methods in identifying anxiety and depressive symptoms and was associated with reductions in clinician-rated outcomes over time. The findings illustrate the potential of continuous digital monitoring for mental health assessment while highlighting challenges in modelling more complex symptom patterns.

Wanniarachchi et al. conducted a scoping review examining how personalization is implemented in digital mental health interventions for adolescents and young people with anxiety and depression. The review found that personalization was often limited in scope, with many interventions using only a single personalization dimension, most commonly the user choice of therapeutic content. While some studies employed machine learning approaches, generative AI was absent, highlighting opportunities for further development in personalized digital mental health interventions.

Prevention remains a critical priority in youth mental health, particularly given the early onset of many mental health conditions. Di Pierdomenico et al. conducted a scoping review of universal (Tier 1) digital mental health interventions for children and youth aged 0–18 years. The review identified widespread use of psychoeducation and cognitive behavioural approaches, while highlighting key gaps including limited representation of early childhood, insufficient equity reporting, and minimal youth involvement in intervention design.

Löchner et al., present a multidisciplinary hypothesis and theory paper outlining future directions for preventive mental health strategies during adolescence. Drawing on expertise from fields including psychology, computer science, physical activity, nutrition, economics, and policy, the authors propose holistic and individualized prevention approaches supported by AI-enabled mHealth tools and digital biomarkers. They emphasize the importance of participatory research, cost-effectiveness evaluation, and integration within existing healthcare systems.

Baumann et al., conducted a systematic review and meta-analysis examining mobile health (mHealth) interventions targeting physical activity, sedentary behaviour, nutrition, and sleep among adolescents with emotional, behavioural, and eating disorders. The analysis found modest but significant improvements in anxiety and eating disorder symptoms, while effects for depression and behavioural disorders were mixed. Interventions with higher session frequency, greater intensity, longer duration, and hybrid delivery formats (e.g., combining digital components with face-to-face support) tended to show larger effect sizes, although these features may limit scalability.

Together, these contributions highlight the potential of digital tools in preventive care while emphasising the need for stronger evidence and broader inclusion of diverse youth populations.

Another important theme emerging from this collection is the role of digital tools in improving mental health literacy and facilitating help-seeking among young people. Vicary et al., examined the co-adaptation of a digital mental health literacy intervention for young people aged 12–14 in the United Kingdom. Using experience-based co-design with young people, parents, and professionals, the study culturally adapted an existing programme and evaluated its feasibility through a cluster-randomized pilot trial. The findings indicate good acceptability while highlighting practical considerations for recruitment, data collection, and engagement in future large-scale evaluations.

Oti et al., evaluated the usability of the Pathway prototype, a digital tool designed to help university students identify appropriate mental health support. Across three cycles of usability testing with students, experts, and stakeholders, participants reported that the platform provided a quicker, more trustworthy, and more private way to search for support compared with existing approaches. Together, these studies highlight the importance of designing digital tools that improve mental health literacy and reduce barriers to help-seeking among young people.

Beyond intervention development, several studies emphasise the importance of robust theoretical frameworks and implementation strategies for digital mental health technologies.

Cowling et al., describe the development of a Theory of Change to guide the design and evaluation of a digital mental health platform for young people. Following a mixed-methods process involving youth, service staff, and external experts, the authors developed a framework linking platform features to intended outcomes and offer recommendations for designing user-centered digital interventions.

Tibbs et al., conducted a theory-driven evaluability assessment of Jigsaw Live Chat, an Irish single-session synchronous chat-based mental health support service for young people. Using situational analysis, literature review, internal data review, and co-design workshops with staff, the authors identified contextual factors, core components, mechanisms of change, and intended outcomes, which informed a causal programme theory and tailored indicators for evaluating this drop-in digital service.

Gee et al., conducted multi-stakeholder research examining the implementation of digital therapeutic interventions within children and young people's mental health services in the United Kingdom. The study identified strong support for digital approaches but also highlighted barriers to implementation, including integration within clinical pathways, the need for strengthened leadership and guidance, and sustained funding.

Dulsen et al., report lessons from an attempted randomized controlled trial evaluating an internet- and mobile-based intervention for adolescents aged 12–18 with mentally ill parents. Recruitment challenges prevented meaningful analysis, highlighting the difficulty of engaging this population and suggesting that future digital interventions might benefit from more family-inclusive approaches.

These studies demonstrate that successful digital mental health innovation requires careful attention to implementation processes and system-level integration.

The collection also highlights emerging digital approaches to delivering and understanding mental health support for young people. Keyan et al., describe the development and cultural adaptation of a World Health Organization transdiagnostic chatbot designed to support adolescents and young adults experiencing psychological distress in low- and middle-income settings. Developed using a human-centered design approach with input from young people, community members, and experts, the chatbot addresses common problems such as low mood, stress, and anger through structured conversational interactions. The intervention is currently undergoing randomized evaluation.

McAlister et al., conducted a retrospective analysis examining associations between children's screen media use and mental health symptoms within a pediatric digital mental health intervention. Elevated screen use was associated with greater baseline anxiety, depressive symptoms, and behavioral difficulties, while reductions in screen use during care coincided with improvements in some symptoms.

## 3 Summary

The 13 articles in this Research Topic highlight an evolving landscape in digital mental health research for youth. Advances in AI-driven measurement, personalization, chat-based support, digital interventions, and theory-informed evaluation demonstrate growing innovation in the field. At the same time, persistent challenges related to equity, implementation, and youth involvement underscore the need for continued research and collaboration. Ensuring meaningful youth participation in the design, evaluation, and implementation of digital mental health interventions remains a central priority. As Topic Editors, we hope this collection encourages further interdisciplinary collaboration and participatory research. The future of digital mental health for youth will depend not only on technological innovation but on our ability to embed these advances within ethical, equitable, and developmentally responsive systems of care. There are many important research directions that warrant further exploration, including the impact of GenAI/LLMs on mental health and wellbeing, how young people are currently using LLMs, and how potential risks and harms can be mitigated.
